# Oxygen doping of HTSC and resistive switching in HTSC-based heterostructures

**DOI:** 10.1186/2193-1801-2-384

**Published:** 2013-08-15

**Authors:** Natalia A Tulina, Ivan Yu Borisenko, Andrey A Ivanov, Andrey M Ionov, Ivan M Shmytko

**Affiliations:** Institute of Solid State Physics RAS, Chernogolovka, Russia; Institute of Microelectronics Technology and High Purity Materials RAS, Chernogolovka, Russia; National Research Nuclear University “MEPhI”, Moscow, Russia

## Abstract

**Abstract:**

The studies of the bipolar resistive switching effect in thin film heterojunctions (YBa_2_Cu_3_O_7−*δ*_/Ag) and (Nd _2−*x*_Ce_*x*_CuO_4−*y*_/Ag) have exhibited the role of oxygen as a doping element in hole- and electron-doped HTSC compounds.

## Introduction

Most HTSC materials are hole-doped compounds. Nd_2−*x*_*Ce*_*x*_*CuO*_4−*y*_ (NCCO), Pr_2−*x*_*Ce*_*x*_*CuO*_4−*y*_ (PCCO), as well as Ba_1−*x*_*K*_*x*_*BiO*_3−*y*_ and BaPb_1−*x*_*Bi*_*x*_*O*_3−*y*_ are few representatives of electron-doped ones. The hole- and electron-doped compounds differ by their physical properties and their phase diagrams (Dagotto 1994). Nevertheless, they have a feature in common. It is strong dependence of the properties on oxygen stoichiometry, since oxygen plays significant role in formation of the basic (ground) state in these compounds. Influence of oxygen is well studiled for p-type HTSC, it is important as hole dopant. However, questions still remain concerning the role of oxygen in electron-doped HTSC cuprates (Orenstein and Wishwanath 2010). The studies of Hall effect (Jiang et al. 1994; Mao et al. 1995) in oxygen-reduced NCCO and PCCO revealed presence of holelike states in these n-type materials. The question arises whether the presence of p-type carriers is crucial for superconductivity in HTSC.

The effect of bipolar resistive switching (BRS) observed in oxide-based heterostructures, including based on strongly correlated electron systems (SCES), such as HTSC (see, for instance, (Waser and Aono 2007; Tulina 2007; Sawa 2008; Kang et al. 2007;Yang et al. 2008; Inoue et al. 2008)), is sensitive to type of charge carriers in SCES. BRS is exhibited in SCES-normal metal heterocontacts at specific polarity of the electric field as a change of the phase composition of the SCES surface layer at the nanosize level. As a result, the metastable high resistance (OFF) and low resistance (ON) states of the heterocontact are realized leading to colossal electrical resistance (CER)(Tulina 2007). CER is the ratio of the resistance on OFF state to the resistance in ON state and characterizes the memory effect. CER = ΔR/R = (R_*OFF*_(V = 0) − *R*_*ON*_(V = 0))/R_*ON*_(V = 0). Intensive fundamental and application studies of this phenomenon are underway since BRS-based devices are regarded as a new generation of alternative nonvolatile memory (Meijer 2008). Besides, BRS effect allows to study the microscopic nature of SCES underlying such devices.

Recent studies of the BRS effect in various compounds revealed the essential role of the oxygen diffusion processes. Electric field of different polarity induces two resistive states of the degraded HTSC surface which is indicative of its separation into conducting and dielectric phases. The switching process gives rise to metastable states with different oxygen content in the interface area of the compound. It is this area that determines the resistive properties of the structure (Tulina et al. 2001; Tulina and Sirotkin 2004). Therefore, the BRS effect involves reproducible oxygen doping of a specific sample area. In (Tulina and Klinkova 2007) we observed BRS inversion in heterojunctions based on electron-doped BaK_0,6_*Bi*_0,4_*O*_3−*y*_: the switching to the high resistance metastable phase takes place when the HTSC crystal has a negative potential with respect to the normal electrode. In this case the current field (*J* = *σE*) is directed to the surface in contrast to switching observed in hole-doped compounds.

## Experiment

**Figure 1:**
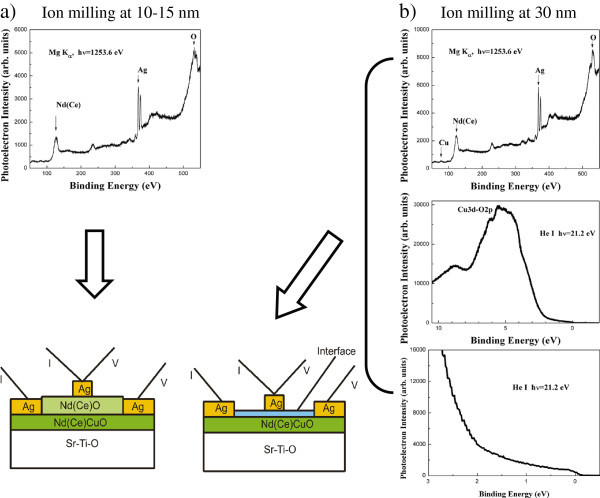
**XPS and UPS of the as grown and ion milled Nd**_**1.75**_***Ce***_**0.15**_***CuO***_**4−*y***_**films.** **(a)** Initial stage of ion milling (up to 10-15 nm depth). XPS spectrum of the film surface (top), corresponding heterocontact scheme (bottom); **(b)** Ion milling at 30 nm depth. XPS spectrum (top) and UPS spectra (middle and bottom) together with the heterocontact scheme.

The aim of the work was to observe bipolar resistive switching effect in YBa_2_Cu_3_O_7−*δ*_/Ag and NCCO/Ag heterojunctions in order to trace the role of oxygen as doping element in p-type and n-type HTSC. Thin epitaxial films of YBCO(001) and NCCO(001) grown by pulse laser deposition were used to produce HTSC-based heterostructures. Figure 1 shows a heterocontact scheme. Silver counterelectrodes were deposited on the as-grown surface to form a HTSC film/interface/Ag structure. The upper silver electrode was deposited through a mask as point electrodes 1 mm in diameter. The electric supply leads were attached to the silver contacts with conductive paste or a mechanical contact in a micromanipulator. The x-ray studies of the YBCO films has revealed that, besides the basic composition, sample contains surface regions with a smoothly decreasing oxygen content down to *δ* = 1,0 (Jorgensen et al. 1990).

Results of NCCO films structure study were reported elsewhere (Tulina et al. 2011). There were detected reflexes of NCCO and Nd_0.5_*Ce*_0.5_*O*_1.75_(NCO) phase. Both phases were epitaxial and (001)-oriented. The obtained results are consistent with other evidences of two phases coexistence after oxygen reducing even in perfect single crystals (Mang et al. 2004). Along with the main phase, the second phase can affect the behavior of the interface of the studied heterojunctions and lead to changes in the type of conductivity. Photoemission studies provided support for the X-ray data. After the film surface was milled with nitrogen ions at depth of 50 nm, it was shown using X-ray photoelectron spectroscopy (XPS) and ultraviolet photoelectron spectroscopy that the film surface was metallic and had an entire set of elements (Figure 1). We thus obtained heterojunctions of two types on NCCO films: (a) with a surface buffer layer formed by NCO (Figure 1a; see the XPS data above) and (b) without the NCO oxide layer, which was removed by ion milling to the main phase NCCO under photoemission control (Figure 1).

**Figure 2:**
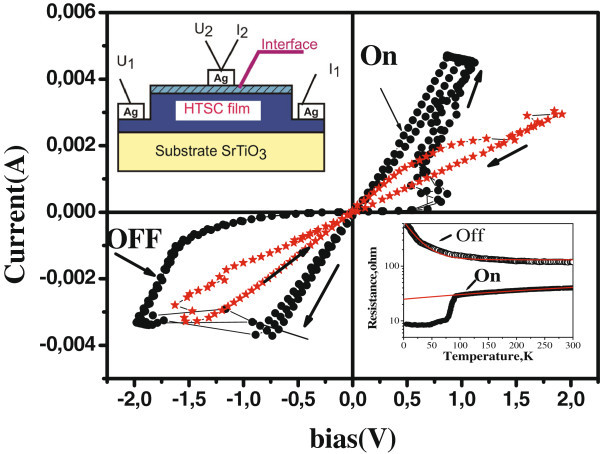
**Examples of the voltage-current characteristics of the YBa**_**2**_***Cu***_**3**_***O***_**7−*δ***_**/Ag (black points) and Nd**_**1,75**_***Ce***_**0,15**_***CuO***_**4−*y***_ **/ Ag (red points) heterocontacts with resistive switching.***T* = 300 K. Upper left corner - heterocontact scheme; Lower right corner - temperature dependences of YBa_2_Cu_3_O_7−*δ*_/Ag heterojunction resistance in Off and On states.

The value of the BRS effect was insignificant in the heterocontacts formed immediately after the ion milling of NCCO surface, the switching pattern corresponded to the electron-doped systems (see Figure 2). This fact can be understood considering weak influence of oxygen as a doping element on the properties of NCCO which has been already confirmed in some works (Higgins et al. 2006;Gauthier et al. 2007). Nonstoichiometric oxygen does not affect the number of carriers in NCCO and leads to disorder only. At the same time the BRS effect was considerably stronger in the YBCO/Ag heterocontacts, and the switching pattern corresponded to the hole-doped systems (Figure 2). As it is seen from inset in the Figure, superconducting transition is observed in the On state. It means that the electric field has induced oxygen diffusion into the controlled region and the Tc value is close to optimal one for YBCO.

## Conclusions

- The bipolar resistive switching has been experimentally observed in NCCO- and YBCO-based film heterojunctions.
- It has been shown that the surface layer of the studied HTSC films is oxygen degraded and can serve as an interface in HTSC perovskite-based heterostructures to create memory elements based on resistive switching.
- The switching polarity and the BRS effect value in the electron- and hole-doped compounds reflect the pattern of their oxygen doping.
